# Use of reverse angle guide catheter with trans-radial approach in patients undergoing middle meningeal artery embolization

**DOI:** 10.3389/fneur.2022.990722

**Published:** 2022-10-25

**Authors:** Neeharika Krothapalli, Mohamad Fayad, Smit Patel, Ahmed Elmashad, Eric Sussman, Charles Bruno, Andrew Grande, Bharathi Jagadeesan, Brendan Killory, Mark Alberts, Inam Kureshi, Martin Ollenschleger, Ramachandra Tummala, Tapan Mehta

**Affiliations:** ^1^Department of Neurology, University of Connecticut, Farmington, CT, United States; ^2^Department of Interventional Neuroradiology, Hartford Hospital, Hartford, CT, United States; ^3^Department of Neurosurgery, Hartford Hospital, Hartford, CT, United States; ^4^Department of Neurology, Radiology and Neurosurgery, University of Minnesota, Minneapolis, MN, United States; ^5^Department of Neurology, Hartford Hospital, Hartford, CT, United States

**Keywords:** reverse angle guide catheter, trans-radial approach, middle meningeal artery, embolization, subdural hematoma

## Abstract

**Background:**

Trans-radial access (TRA) for MMA embolization has grown due to lower access site complications and greater patient satisfaction. Here, we describe the feasibility of utilizing a 6F Envoy Simmons 2 (6F-SIM2) as a guide catheter with TRA and compare outcomes with trans-femoral approach (TFA) in a single center case series.

**Methods:**

We performed a retrospective review of patients who underwent MMA embolization for management of chronic subdural hematoma (cSDH). TRA was performed by utilizing a combination of 6F 90cm Envoy (Codman & Shurtleff, Inc., Rayham, MA) Simmons 2 guide catheter and 5F 125cm Sofia (Microvention, Aliso Viejo, CA) intermediate catheter. Outcomes measured are Modified Rankin Score (mRS) at 90 days, inpatient mortality, post-embolization recurrence, fluoroscopy time and radiation exposure.

**Results:**

A total of 71 patients underwent 97 MMA embolization overall with 65 (67%) in trans-femoral access group, 11 (11.3%) in trans-radial access without use of Simmons 2 Guide catheter group and 21 (21.6%) in trans-radial access with use of Simmons 2 Guide catheter group. There were no direct access-related complications in either group. One patient had thromboembolic stroke in trans-femoral group. There was no difference in average procedure-related total fluro time or radiation dose among all three groups.

**Conclusion:**

Trans-radial approach using 6F-SIM2 guide catheter coupled with 5F Sofia intermediate catheter is safe and effective. It provides an alternative approach to access distal branches of bilateral anterior circulation in elderly patients with difficult anatomy undergoing MMA embolization.

## Introduction

The feasibility of trans-radial approach has been demonstrated in a variety of endovascular procedures including flow diversion, aneurysm coiling, carotid stenting, acute ischemic stroke and MMA embolization (1, 2) for its lower rate of access complications and reduced cost (3, 4). Most importantly, trans-radial approach (TRA) has a much higher patient satisfaction due to earlier ambulation, shorter hospital stay, and avoidance of groin shaving and exposure. Several studies did directly elucidate the differences in the angiographic and clinical outcomes of TRA and trans-femoral approach (TFA) (5, 6). In patients with a bovine aortic arch and common carotid innominate trunk, TRA with a 6F larger system for intracranial embolization is less challenging compared to the more common non-bovine aortic arch as there is lesser likelihood of requiring a Simmons shape catheter for shape formation within the aortic arch. Moreover, catheterization of the left middle meningeal artery through trans-radial approach can be challenging especially in non-bovine Type II/III aortic arches. The risk of guide catheter herniation remains higher in Type II/III arches with pre-existing tortuosity in great vessels. Unstable access does theoretically increase the risk of complications during intracranial embolization. This can be averted by utilizing a guide catheter with Simmons2 shape, which can redirect intermediate and microcatheter force during advancement into the carotid artery as the curvature point of a Simmons catheter is supported by the ventral wall of an aortic arch. Previous studies have described use of reverse angled guide catheter use for trans-femoral and trans-radial approach for treatment of carotid artery stenting (7–9).

In our study, we describe the feasibility of tri-axial trans-radial approach using 6F Envoy Simmons 2 (6F-SIM2) guide catheter coupled with a 5F Sofia intermediate catheter to perform bilateral MMA embolization.

## Methods

This is a single center retrospective observational study of patients age ≥18 with cSDH who presented to our comprehensive stroke center between 01/01/2020 to 12/01/2021 and underwent MMA embolization procedures for cSDH. MMA embolization was performed on cSDH patients with CT head demonstrating iso-density or hypodensity of ≥50% of the volume of blood products in the subdural space. Patients also had to have evidence of mass effect with or without midline shift. They required a 24-h follow-up head CT indicating stability of the SDH. Patients with neurological symptoms such as headaches, cognitive decline, speech difficulties, gait impairment or imbalance, weakness, paresthesia, and seizures who were not surgical candidates were not excluded from an MMA embolization. Patients were excluded from MMA embolization if they required urgent neurosurgical evacuation, had focal SDH within frontal, temporal base or interhemispheric space without involvement of the cerebral convexity, demonstrated secondary SDH due to vascular lesion such as dural arteriovenous fistula or arterial venous malformation, brain tumor, spontaneous intracranial hypotension or in setting of craniotomy not performed for subdural evacuation. Patients with comorbidities such as cardiopulmonary instability, severe carotid artery disease resulting in occlusion, near occlusion of the external carotid artery or increasing risk of stroke, mRS of 4–6 or life expectancy < 1-year, end stage renal disease and significant coagulopathy were excluded. Pregnant patients or patients with contralateral blindness were also excluded. Access approach was determined by the proceduralist. Study variables included age, gender, vessel anatomy (aortic arch type, left common carotid artery loop, brachiocephalic trunk tortuosity), side of SDH, catheter system used, type of embolic material, fluoroscopy time, radiation dose, etiology of hematoma, anticoagulants or antiplatelets used, history of coagulopathy, presence of midline shift and size of pre-embolization SDH on initial head CT as reported by the radiologist. Focused procedure-related outcomes measured failed access, guide catheter herniation events, access site complications and peri-procedural ischemic stroke.

### Statistical analysis

A descriptive analysis was performed for all patients with MMA embolization of cSDH and compared TFA vs. TRA without use of Simmons 2 Guide catheter vs. TRA with use of Simmons 2 Guide catheter groups. Wilcoxon Rank-Sum test and Student's t-test for continuous variables and categorical variables were compared using Chi-square tests of proportion as cell frequencies permitted and Fisher's exact test when frequencies were ≤ 5. Comparison was univariate as small sample size was insufficient for multivariate analysis. Data analysis was performed with statistical package SAS 9.4. This study was approved by the Institutional Review Board of our hospital with total waiver of consent since it does not contain direct enrollment of patients.

### Procedural description for cases with use of 6F-SIM2 guide catheter

**Figure 1:**
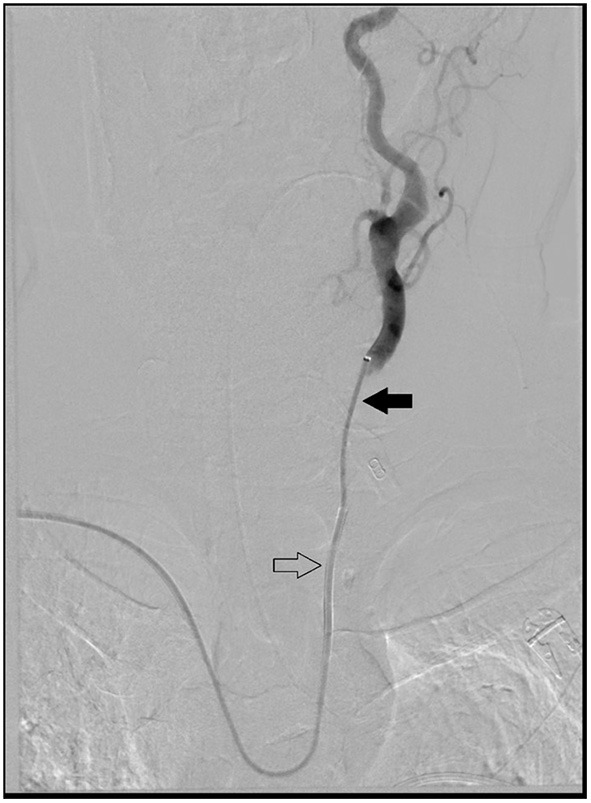
Anterior-posterior view of head and neck demonstrating 6F Envoy Simmons2 access with 5F Sofia intermediate catheter with left common carotid artery run.

**Figure 2:**
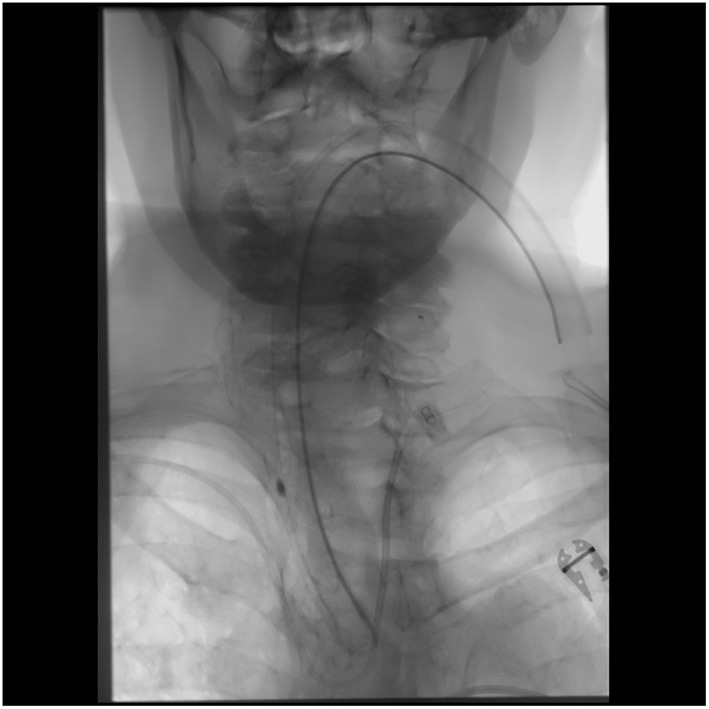
Anterior-posterior view of head and neck showing catheterization of left common carotid artery.

All procedures were performed under general anesthesia. Access to right radial artery was obtained under ultrasound guidance and a 6F slender Terumo radial sheath was placed and connected to continuous flush of heparinized saline at a concentration of 5iu/ml. A 6F Envoy Simmons 2 guide catheter as well as a 5F Sofia intermediate catheter were flushed, prepped on a sterile table and connected to continuous flush of heparinized saline. The intermediate catheter was introduced into the guide catheter followed by a baby J Glide wire into the intermediate catheter. The system was then placed into the radial sheath and baby J Glide wire was introduced about 30 cm followed by 10 cm of the intermediate catheter. Subsequently, the entire system was advanced together under fluoroscopy guidance into the descending aortic arch. The Glide wire was then retrieved and intermediate catheter was pulled back proximal to the Simmons 2 shape. In select cases with Type III anatomy, it can be difficult to navigate the glide wire over the aortic arch. In these situations, we typically used a 5F 120 cm Angled tip catheter to facilitate ascent of the glide wire over the lesser curve of the aortic arch. Next, the guide catheter was slowly rotated in a counterclockwise maneuver until the Simmons knot is formed and advanced into the ascending arch. After the common carotid artery was selected, the Sofia intermediate catheter was advanced over a glide wire under roadmap guidance to properly select the internal carotid artery for orbital blood supply confirmation. The external carotid artery was then selected. A variety of microcatheters were used for embolization based on compatibility with the form of embolization. Figure 1 is an anterior-posterior view of head and neck demonstrating 6F Envoy Simmons2 access (hollow arrow) with 5F Sofia intermediate catheter (solid arrow) with a left common carotid artery run. Figure 2 is an anterior-posterior view of head and neck without digital subtraction angiography and shows catheterization of the left common carotid artery.

## Results

A total of 71 patients underwent a total of 97 MMA embolization with 65 (67%) in trans-femoral access group, 11 (11.3%) in trans-radial access without use of Simmons 2 Guide catheter group and 21 (21.6%) in trans-radial access with use of Simmons 2 Guide catheter group. Table 1 demonstrates baseline clinical characteristics and outcomes of the case series. There was no difference observed in anatomical factors contributing to difficult access like tortuosity of left common carotid artery [TFA (41.5%), TRwoSim2 (45.5%); *p* = 0.098 vs. TRwSim2 (57.1%); *p* = 0.20], tortuous right subclavian-brachiocephalic trunk [TFA (75.4%), TRwoSim2 (72.7%); *p* = 0.85 vs. TRwSim2 (71.4%); *p* = 0.72], Type II/III arch [TFA (Type II 95.4%, Type III 1.5%), TRwoSim2 (100%); *p* = 0.54 vs. TRwSim2 (Type II 90.5%, Type III 9.5%); *p* = 0.40 and 0.08], or aortic arch diameter > 28 mm [TFA (40%), TRwoSim2 (36.3%), TRwSim2 (42.8%); *p* = 0.82]. We also did not observe any difference among the three groups for radiation dose [TFA (723 mGy), TRwoSim2 (557 mGy); *p* = 0.74 vs. TRwSim2 (672 mGy); *p* = 0.76] or fluoroscopy time [TFA (40.5 min), TRwoSim2 (29 min); *p* = 0.33 vs. TRwSim2 (33.1 min); *p* = 0.22].

**Table 1:** Baseline clinical characteristics and outcomes.

	**TFA,** ***N =* 65** ***N* (%)**	**TRwoSim2** ***N =* 11** ***N* (%)**	***P* value** ** TFA vs.** ** TRwoSim2**	**TRwSim2** ***N =* 21** ***N* (%)**	***P* value TFA vs. TRwSim2**
**Gender**					
Male	43 (66.2)	11 (100.0)	0.098	17 (81.0)	0.20
**Vessel anatomy**					
Left Common Carotid Artery tortuosity	27 (41.5)	5 (45.5)	0.81	12 (57.1)	0.21
Aortic arch >28 mm	26 (40.0)	4 (36.3)	0.82	9 (42.8)	0.82
Tortuous right subclavian-brachiocephalic trunk	49 (75.4)	8 (72.7)	0.85	15 (71.4)	0.72
Type II arch	62 (95.4)	11 (100)	0.54	19 (90.5)	0.40
Type III arch	1 (1.5)	0 (0.0)	–	2 (9.5)	0.08
**Etiology of subdural hematoma**					
Spontaneous	29 (44.6)	5 (45.5)	0.96	8 (38.1)	0.60
**Anticoagulation/Antiplatelet use**					
Anticoagulation at time of index event	19 (29.2)	2 (18.1)	0.45	9 (42.9)	0.25
Antiplatelet agent at time of index event	19 (29.2)	7 (63.6)	0.026	4 (19.0)	0.36
**Type of embolic material**			< 0.001		< 0.001
Particle	54 (83.1)	0 (0.0)		0 (0.0)	
Liquid Embolic	10 (15.4)	8 (72.7)		21 (100)	
**Continuous variables (median Q1-Q3)**					
Age (years)	73 [69–84]	64 [60–77]	0.046	74 [59–78]	0.27
**Radiation variables**					
Fluoroscopy time (minutes)	40.5 [31.6–51.5]	29.0 [27.4–55.8]	0.33	33.1 [22.9–38.2]	0.22
Radiation dose (mGy)	723 [485.7–1,301]	557.05 [493.9–759.8]	0.74	672 [566.6–1,079]	0.76
Radiation dose (mcGym sq)	8,738.5 [6,099.5–16,321]	6,291.5 [5,810–9,016]	0.83	8,418.5 [7,552–12,656]	0.96
**Subdural hematoma size (mm)**					
Size of subdural hematoma at embolization time	12 [8-17]	11 [6-15]	0.54	11 [7-16]	0.43
Subdural hematoma follow up (days)^*^					
Time Anticoagulation resumed	14 [9-56]	118 [118–118]	0.06	65.5 [26–105]	0.14
Time Antiplatelet resumed	14 [3-100]	92.5 [72–113]	0.034	28 [28–28]	0.90
**Outcomes**					
Recurrence post embolization	2 (3.1)	0 (0.0)	–	0 (0.0)	–
Mortality at 90 days unrelated to procedure	3 (4.6)	1 (9.0)	0.54	0 (0)	–
Mortality related to procedure	0 (0)	0 (0)	–	0 (0)	–

* Reported values are based on available follow-up data.

There was no difference observed in anticoagulation use at time of index event [TFA (29.2%), TRwoSim2 (18.1%), TRwSim2 (42.9%)]. Antiplatelet use at time of index event was higher in TRwoSim2 group compared to TFA group (63.6 vs. 29.2%; *p* = 0.026). A significant variability was noted in resumption of antiplatelet or anticoagulant medications (as reported in Table 1). The projected time for antiplatelet or anticoagulation hold was not available due to variations in decision making in individual clinician plans and complexity of individual patient case. Determining the role of antiplatelet or anticoagulation use contributing to recurrence of subdural hematoma is beyond the scope of this study.

Mortality unrelated to procedure occurred in 3 (3.9%) patients overall with 1 (3.6%) in TRA and 2 (4.17%) in TFA group within 90 days. There was no mortality related to procedure in either group. There were no failed cases that necessitated a different set-up. There were no access site complications in either group but one patient had thromboembolic stroke in trans-femoral group. Two patients in trans-femoral group had guide catheter herniation at the time of MMA catheterization.

## Discussion

Trans-radial access (TRA) for coronary interventions demonstrated >60% reduction in access site complications (10–12). Utilization of TRA for neuro-interventional procedures provides certain advantages over trans-femoral approach (TFA) including immediate ambulation, greater patient satisfaction, reduced postprocedural hospital stay and cost saving (13–15). This strategy has now been employed in a variety of neuro-interventions including diagnostic angiograms, flow diversion, mechanical thrombectomy, aneurysm coiling, AVM embolization and carotid artery stenting (5, 6, 16, 17).

Superiority of radial vs. femoral vascular access can be difficult to assess in patients with ischemic stroke, intracranial aneurysm embolization or high flow vascular malformation embolization. The variation in selection of ideal access approach is significantly influenced by necessity of a larger size guide catheter and/or speed to approach the target lesion (i.e., in mechanical thrombectomy cases with ischemic stroke). One of the major strengths of our study is that we have compared the outcomes of access approaches for only elective MMA embolization cases, which usually does not require larger than 6F access. The patient population is also more uniform as the majority of MMA embolizations are common in elderly patients (median age of 73 years) with higher incidence of arch complexities. At our organizations, individual operators had different access route preferences. However, those who used radial first as the MMA embolization approach prefer to use 6F Envoy transradial access set up as primary approach as long as radial artery anatomy is favorable. For trans-radial approach, the only exception for not using reverse angle guide catheter was bovine arch anatomy as it can be easily accessed with straight tip or angled tip catheters and does not require additional support. Traditionally we have also avoided transradial approach with heavy calcifications involving aortic arch or right subclavian-brachiocephalic complex as the risk of plaque disruption would be theoretically higher with manipulation of reverse angle guide catheters.

In literature, TRA has demonstrated maximal benefit in patients with bovine arch, large body habitus, occlusive aorto-iliac disease, approach to posterior circulation and concurrent use of anticoagulation or antiplatelet agents during procedure (18). Despite the plethora of benefits with a trans-radial approach, there are some hurdles to overcome. Some unfavorable factors for radial artery access include Type II or III arch while selecting intracranial segments of left internal or external carotid artery branches, marked subclavian or innominate tortuosity, small size of radial artery and other uncommon arch variants. Jo et al reported 82.6% success rates of selective catheterization to the left internal carotid artery through radial access (19). A prospective review by Sattur et al demonstrated that procedural success was achieved in 92.3% of patients who underwent TRA and crossover to femoral route occurred in 6.5% of cases (20). In our series, there were no access-site complications or TRA-to-TFA conversion.

Our study has several limitations. The retrospective design of our study may have led to selection bias, limiting the generalizability of results. In this analysis, there is data for patients treated with MMA embolization but not for proportions of patients excluded by the initial triage process. Small sample size and individual operator bias for procedural methods are significant confounders. Determining the role of antiplatelet or anticoagulation use contributing to recurrence of subdural hematoma is beyond the scope of this study.

In summary, our study demonstrates that utilizing a reverse angle guide catheter like 6F 90 cm Envoy (Simmons 2 shape) along with soft 5F Sofia intermediate catheter in type II/III arches may be a relatively safe and proficient approach to access distal left anterior circulation branches through TRA. The 5F Sofia distal access catheter is soft enough that it does not disrupt the Simmons 2 shape of 6F Envoy guide catheter. This approach may be superior to TFA in select procedures like MMA embolization, where TFA with tortuous aortic arch may prove more time consuming, difficult and demonstrate higher embolic risk in atherosclerotic vessels (21).

## Data availability statement

The raw data supporting the conclusions of this article will be made available by the authors, without undue reservation.

## Ethics statement

The studies involving human participants were reviewed and approved by Hartford Hospital, HHC-2021-0088. Written informed consent for participation was not required for this study in accordance with the national legislation and the institutional requirements.

## Author contributions

NK and TM: substantial contribution to conception, design, analysis and interpretation of data, drafted and reviewed the work. MF and SP: drafted and reviewed the work. AE, ES, CB, AG, BJ, BK, MA, IK, MO, and RT: reviewed the work. All authors contributed to the article and approved the submitted version.

## Conflict of interest

The authors declare that the research was conducted in the absence of any commercial or financial relationships that could be construed as a potential conflict of interest.

## Publisher's note

All claims expressed in this article are solely those of the authors and do not necessarily represent those of their affiliated organizations, or those of the publisher, the editors and the reviewers. Any product that may be evaluated in this article, or claim that may be made by its manufacturer, is not guaranteed or endorsed by the publisher.
